# Infectivity and RNA Persistence of a Norovirus Surrogate, the Tulane Virus, in Oysters

**DOI:** 10.3389/fmicb.2018.00716

**Published:** 2018-04-12

**Authors:** David Polo, Julien Schaeffer, Peter Teunis, Vincent Buchet, Françoise S. Le Guyader

**Affiliations:** ^1^Laboratoire de Microbiologie, Laboratoire Santé, Environnement et Microbiologie-Santé, Génétique et Microbiologie des Mollusques, Institut Français de Recherche pour l’Exploitation de la Mer (IFREMER), Nantes, France; ^2^Hubert Department of Global Health, Emory University, Atlanta, GA, United States; ^3^Laboratoire Sécurisation des Productions en Conchyliculture/Santé, Génétique et Microbiologie des Mollusques, Institut Français de Recherche pour l’Exploitation de la Mer (IFREMER), Bouin, France

**Keywords:** infectious virus, genome detection, oysters, persistence, shellfish outbreaks

## Abstract

Oysters, being filter feeders, can accumulate some human pathogens such as norovirus, a highly infectious calicivirus, most common cause of acute gastroenteritis worldwide. Accumulated virus decays over a period of days to weeks, possibly rendering contaminated oysters safe again. Sensitive molecular methods have been set up for shellfish analysis but without answering the question of infectious virus detection. Using the Tulane virus (TV), a norovirus surrogate that recognizes the same ligand as human norovirus in oyster tissues, the genome and infectious virus decay rates were estimated using inverse linear regression in a Bayesian framework for genome copies. Infectivity decreased faster than genome copies but infectious viruses were detected for several days. Quantifying the decrease in viral infectivity and genome detection in oysters over such a long period may help local authorities to manage production areas implicated in shellfish-borne outbreaks, and thus protect consumers.

## Introduction

Shellfish, being filter feeders, are known to accumulate different types of pathogens of human fecal pollution. As a consequence, they have been implicated in many gastroenteritis outbreaks, and norovirus (HuNoV) is the agent most frequently detected following oyster consumption (EFSA, 2012; Yu et al., 2015). Several factors contribute to these outbreaks. First, among the human enteric viruses, HuNoVs are recognized as the leading cause of epidemics and sporadic cases of gastroenteritis in all age groups (De Graaf et al., 2016). Additionally, HuNoVs are excreted in large quantities by ill people as well as by asymptomatic individuals (Teunis et al., 2015). Given the foregoing, huge numbers of HuNoV particles are discharged in sewage and due to their resistance to inactivation, they are frequently detected in wastewater treatment plant effluent and in surface waters (Pouillot et al., 2015; Kazama et al., 2016). Second, depuration of shellfish, which was developed to eliminate bacteria, does not efficiently eliminate viruses, which can persist for several weeks or months in bivalve tissues (McLeod et al., 2017). The identification of specific ligands for human HuNoV in various oyster species may contribute to this long persistence (Le Guyader et al., 2006, 2012; Tian et al., 2007).

Several studies have clearly demonstrated the slow decrease of HuNoV concentrations in oyster samples using genome detection (Dore et al., 2010). But how long infectious virus persists remain unclear. Until recently HuNoV was not cultivable, however, a newly described culture system based on enteroids offers now the capacity to detect infectious HuNoV (Ettayebi et al., 2016). This recent development would help to solve the question of whether a surrogate virus can be identified that correctly mimics the behavior of HuNoVs, and to validate alternative method aiming to detect capsid integrity (Randazzo et al., 2018). Selection of an appropriate surrogate requires consideration of several issues, such as environmental persistence, physical and genetic similarities, ease of propagation, and tissue binding properties (Kniel, 2014). We previously demonstrated the potential of Tulane virus (TV), a member of the recovirus genus (ReCV) of the *Caliciviridae* family, to mimic HuNoV behavior in oyster tissues as it showed the same tissue distribution and a comparable decrease of genomic copies over time (Drouaz et al., 2015).

The objectives of the present study were:

• to quantify the decrease of TV genome copies as inferred from real-time RT-PCR (*r*RT-PCR) and the decrease of TV infectious particles over time in oysters reared in a scientific oyster farm; and
• to evaluate the measurement error estimated from standard curve data, by using inverse linear regression in a Bayesian framework to more accurately assess the decay rate for genome and infectivity detection.

## Materials and Methods

### Viruses and Cell Lines

Mengovirus (MgV) strain pMC0 (provided by A. Bosch, University of Barcelona, Spain) was propagated in HeLa cells, and TV strain M033 (provided by T. Farkas, Louisiana State University at Baton Rouge, LA, United States) in LLC-MK2 cells (ATCC CCL-7; ATCC, Manassas, VA, United States), as previously described (Martin et al., 1996; Farkas et al., 2008). To produce high titer of TV, several large flasks were infected at low multiplicity of infection (MOI). When cytopathic effect (CPE) was complete, cultures were frozen and thawed (-20°C) three times, centrifuged, and the supernatant filtered to 0.22 μm (Minisart^®^, Sartorius, Germany). Viral suspensions were kept frozen at -80°C in aliquots, and titrated by TCID50 or *r*RT-PCR as described below.

### TV Bioaccumulation in Oysters

Four bioaccumulation experiments (named A, B, C, and D) were performed in March, April, May, and early June 2016, each used 250 oysters (*Crassostrea gigas*) directly purchased from the same producer (few days before each experiment). Aquariums were filled with 100 L of natural seawater seeded with 11.3 ± 0.12 log_10_ RNA copies of TV, which corresponds to 7.14 ± 0.1 log_10_ TCID_50_. After 24 h of bioaccumulation, virus concentrations were checked in a random sample of 20 oysters.

### Persistence Experiments

Following bioaccumulation, the oysters were rinsed and transported to an experimental farm with direct access to natural seawater and facilities to maintain oysters for long periods (Drouaz et al., 2015). Oysters were placed in clean seawater in large tanks (500 L) and supplied with constantly circulating aerated and filtered natural seawater (200 L/hour/tank). Parameters such as temperature and salinity were routinely measured. Prior to release into the environment, seawater was treated according to the safety rules of the experimental farm.

### Sample Processing and Virus Recovery

Oysters samples, comprised of 20 individuals each, were collected at time 0 h, 2 and 3 days, and then weekly up to 4 weeks. For the two last experiments (C and D) an additional sampling was performed at day 25. Samples were received at the laboratory in less than 24 h, except for experiment B for which the four first samples were frozen. On arrival, all oysters were weighed, immediately shucked, flesh weight recorded to calculate the allometric coefficient (Drouaz et al., 2015). Then the digestive tissues (DTs) were dissected and distributed into 1.5 g aliquots. One DT aliquot was immediately homogenized in 2 mL of phosphate-buffered saline (PBS, pH 7.4), extracted by vortexing with an equal volume of chloroform-butanol for 30 s, and treated with Cat-Floc T (Calgon, Ellwood City, PA, United States) for 5 min on the bench before centrifugation for 15 min at 13,500 × g. The resulting suspension was precipitated with polyethylene glycol 8000/NaCl (PEG 8000) (Sigma, Saint-Quentin, France) for 1 h at 4°C and centrifuged for 20 min at 11,000 × *g* at 4°C (Atmar et al., 1995).

### Purification for Infectious TV Detection

Then, PEG pellets were suspended with 10 mL of medium M199 (Gibco^®^, Life Technologies) supplemented with antibiotics-antimitotic (Gibco^®^, Life Technologies). An aliquot of 100 μL was kept for nucleic acid (NA) extraction. The mixture was centrifuged at 2862 × *g* for 10 min at 4°C, filtered through a 0.22 μm filter, and concentrated using an Amicon R Ultra-15 (100 K) Centrifugal Filter Device by centrifugation at 5000 × *g* for 20 min at 4°C, to a volume of 1.5 ± 0.2 ml. This concentrate was loaded into PD-10 Desalting columns containing Sephadex G-25 Medium, and equilibrated with M199 medium up to a volume of 2.5 mL, and centrifuged at 1000 × *g* for 2 min. The supernatant was recovered and used for infectivity assays, except for a 100 μL aliquot, which was kept for NA extraction to verify that purifications steps did not induce loss of viral genomic copies.

### Infectivity Assay and Titration Using TCID_50_ Assay

Purified supernatants (1 mL) were inoculated on confluent LLC-MK2 cells in T 75 flasks with 9 mL of M199 medium at 37°C. After 1 h of contact, an aliquot of 100 μL was kept and stored frozen. After incubation for 7 days in an incubator at 37°C with gentle agitation, 100 μl was collected and compared to the 1-h control by *r*RT-PCR. Samples showing an increase of more than three Logs (9 *C*_T_-values) were considered to be positive.

For titration of samples, 1 mL of purified supernatant was serially diluted (10^-1^ to 10^-8^) in medium and inoculated in 10-well for each dilution onto cells in 96-well culture plates (Thermo Fisher Scientific, France) under a volume of 200 μL, and incubated for 7 days at 37°C. For each experiment two 96-well culture plates were inoculated. CPE counts were converted into TCID_50_ units using the Reed and Muench calculation method (Reed and Muench, 1938) and were expressed per g of DT. A negative control was included on each plate.

### NA Extraction and Genome *r*RT-PCR Detection

For each sample, two additional extractions were performed on frozen DT aliquots with MgV added as an extraction control before homogenization. All the first steps were as described as above except that the PEG pellet was recovered in 1 mL of sterile water and NA were extracted immediately.

Nucleic acid extractions were carried out using the NucliSENS kit (bioMérieux, Lyon, France) and an automatic easyMAG extractor (bioMérieux, Lyon, France) according to the manufacturer’s instructions, with minor modifications, as described previously (Le Guyader et al., 2009). NAs were recovered in 100 μL of elution buffer (bioMérieux) and triplicate 5 μL aliquots were analyzed immediately using the UltraSense One-Step quantitative RT-PCR system (Life Technologies, France). For TV, primers TVIFf (5′-CTGGGATACCCACAACATC-3′), TVIFr (5′’-GCCAGTTAACAGCTTCAGC-3′) and probe TVIFp (FAM-TGTGTGTGCCACTGGATAGCTAGCACC-BHQ) were used to amplify a region between nucleotides 3775 and 3884 (GenBank accession number EU391643.1) (Drouaz et al., 2015). MgV spiked into the two additional DT extracts was detected as previously described (Pinto et al., 2009). A standard curve was amplified for each run as previously described (Drouaz et al., 2015).

### Statistical Analysis

Genome and infectivity data were analyzed jointly in a two-level model. In both the infectivity and the genome exponential decay was assumed, using linear regression on the log-transformed numbers of virus particles or genome copies. In the genome model, measurement error was estimated from standard curve data by using inverse linear regression in a Bayesian framework (see Supplementary Material). Error estimates in the infectivity data were used to weight individual infectivity measurements. Numbers of infectious viruses were expressed as a fraction of “total” viruses, as estimated from numbers of genome copies. Hence, the decay of total virus and of the fraction of infectious virus could both be estimated. The two-level model then allowed for estimation of the overall (average) decay rates in total and infectious virus.

The variations in temperature, salinity and allometric coefficient of oysters were compared between the four experiments using ANOVA Fisher and Tukey HSD tests, and were considered significant with a *p*-value of 0.05.

## Results

### Controls and Data Robustness

The oysters used in this study were grown in the natural environment and thus, there may be physiological differences between oyster batches used for each experiment due to small variations in the environmental conditions. To investigate the similarity in physiological state of oyster batches, we measured various environmental parameters and calculated the allometric coefficient (**Table 1**). The mean body weight per oyster and the allometric coefficients were similar in three experiments out of four. For the last experiment performed in June, these values appeared somewhat higher. However, the difference was not statistically significant (*p* = 0.2712 and *p* = 0.1091, respectively). The body weight remained stable over the 4 weeks for all experiments (data not shown). The main difference observed between experiments was water temperature (*p* = 0.0001), and a lower salinity was also noted for experiment A (*p* = 0.0001) (**Table 1**).

**Table 1:** Physical characteristics recorded for the four experiments.

Experiments	Sea water		Oyster		Quality controls	
			
	Temp	Salinity	Body weight	DT weight	allom. coeff	ext. eff
A	9.1 ± 1.6	30.0 ± 1.2^#^	5.86 ± 0.85	0.47 ± 0.09	12.99 ± 3.30	36 ± 6
B	11.4 ± 2.1	31.5 ± 0.8	6.05 ± 0.73	0.47 ± 0.03	12.84 ± 2.00	42 ± 14
C	13.7 ± 2.2	32.3 ± 0.5	5.93 ± 0.50	0.47 ± 0.09	12.55 ± 2.37	36 ± 10
D	17.2 ± 1.8^#^	31.7 ± 1.4	8.29 ± 0.81	0.55 ± 0.11	15.18 ± 2.05	33 ± 10


**The allometric coefficient (allom. coeff) was calculated by dividing the total body weight by the weight of the digestive tissues (DT), and it is expressed here as the arithmetic mean values for all samples analyzed per experiment. The extraction efficiency (ext. eff) represents the mengovirus recovery for each extraction and is expressed as the average value in % obtained for all extracts per experiment. #: statistically different from the other experiments, (p* = *0.0001).**

Mengovirus, added to the two extractions made for genome detection, showed comparable extraction efficiency coefficients, varying from 33 to 42% for all four experiments (**Table 1**).

An additional control was added on the sample prepared for infectivity detection. Indeed to verify that no TV was lost during the purification steps, a portion of the eluate after the Sephadex column was extracted and analyzed by *r*RT-PCR. Thus a total of three NA extracts were analyzed for each sample, and each undiluted NA extract was analyzed by *r*RT-PCR in triplicate (3 × 5 μL).

### *r*RT-PCR Data

The initial virus concentration in oysters was comparable across batches (2.68–4.32 × 10^6^ RNA/g DT); this consistency is important for monitoring and comparing virus decay (**Table 2**).

**Table 2:** Virus concentrations obtained for the four separate experiments as measured by rRT-PCR (RNA genome copies) and infectivity assay (TCID_50_).

Time (Days)	Experiment A		Experiment B		Experiment C		Experiment D	
				
	RNA	TCID_50_	RNA	TCID_50_	RNA	TCID_50_	RNA	TCID_50_
0	4.32 × 10^6^	7.43 × 10^3^	2.68 × 10^6^	*4.18* × *10*^2^	2.85 × 10^6^	1.87 × 10^4^	1.99 × 10^6^	1.32 × 10^4^
2	9.74 × 10^5^	1.48 × 10^3^	8.41 × 10^5^	*18.7*	2.54 × 10^6^	3.72 × 10^3^	No sample	
3	1.48 × 10^6^	1.40 × 10^3^	1.66 × 10^6^	*9*	1.59 × 10^6^	1.87 × 10^3^	2.17 × 10^6^	3.72 × 10^3^
7	4.84 × 10^5^	4.69 × 10^2^	7.45 × 10^5^	*7*	1.04 × 10^6^	3.72 × 10^2^	6.56 × 10^5^	5.90 × 10^2^
14	1.91 × 10^5^	7.43	6.41 × 10^4^	*Positive*	3.91 × 10^4^	4.7	9.95 × 10^4^	Positive
21	1.84 × 10^4^	Positive	8.00 × 10^3^	Positive	4.55 × 10^3^	Positive	1.18 × 10^4^	
25	No sample		No sample		6.38 × 10^3^		4.46 × 10^3^	
28	2.53 × 10^3^		6.63 × 10^2^		3.30 × 10^3^		1.11 × 10^3^	


**RNA: Geometric mean titer calculated based on the concentrations obtained from the three separate extractions and triplicates of rRT-PCR and expressed per g of DT. TCID_*50*_: mean values of two titration plates performed per sample and expressed per g of DT (values in italics for experiment B were derived from samples that were frozen prior to the infectivity test). ‘Positive’ results indicate that some infectious viruses were present but at concentrations too low for titration*.*

**FIGURE 1:**
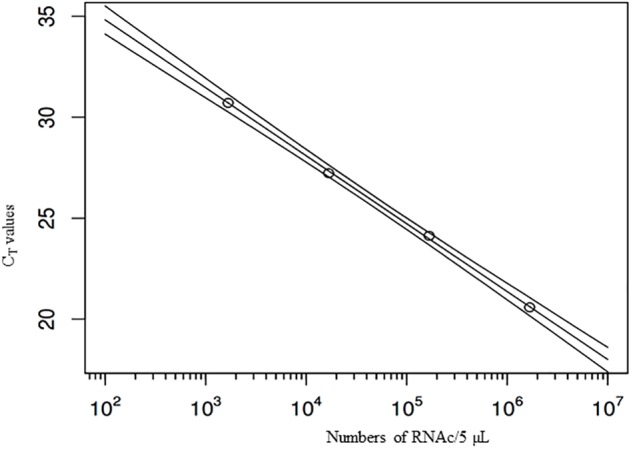
Example standard curve with four dilutions of the stock suspension, and regression line, with (95%) predictive intervals. *X*-axis: numbers of virus genome copies in 5 μl suspension (the outer lines show the prediction intervals), *Y*-axis: *C*_T_-values.

**FIGURE 2:**
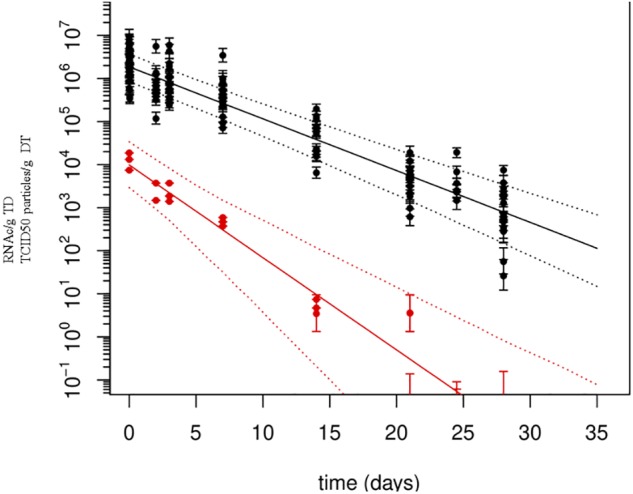
Persistence of Tulane virus (TV) in oysters as determined by genomic detection and infectivity assay. Solid lines represent the mean decay curve and the dotted lines the 95% prediction intervals, as produced by inverse regression analysis. *X*-axis: time (in days), *Y*-axis: numbers of RNA copies or TCID_50_ particles/g of DT.

Quantification using *r*RT-PCR depends on standard curves. To improve error estimation, the Bayesian inverse regression model was used to jointly analyze standard curve data and sample data. This procedure combines the effects of uncertainty in *C*_T_-values and (inverse) regression errors on standard curves, on estimates of numbers of genome copies per g of DT. All standard curves were included in the statistical analysis to account for effects of measurement errors associated with *C*_T_-values. For example, a standard curve which includes the 95% prediction interval for the *C*_T_-values, as estimated in the Bayesian model, shows that the regression error is small over the range of genome copy numbers covered by the standards, but tends to increase when extrapolating outside the range (**Figure 1**). Using these errors for each standard curve, the number of genome copies was calculated for each extraction, based on the three replicate *C*_T_-values (after quality control, verifying that the difference among the three values was lower than 2 *C*_T_). Resulting estimates of genome copy numbers and 95% posterior credibility intervals can be given for all samples analyzed (**Figure 2**). A total of 12 measurements (four experiments quantified three times) were obtained for each sampling time, except for day 25, which was sampled only for the last two experiments. At low virus concentrations, observations are censored (*C*_T_ > 40) and the error estimates increase since confidence in *C*_T_-values between 40 and 45 is limited. Also shown are the overall mean decay curves for all four experiments as predicted by the Bayesian hierarchical model (mean decay and 95% credibility) (**Figure 2**).

**FIGURE 3:**
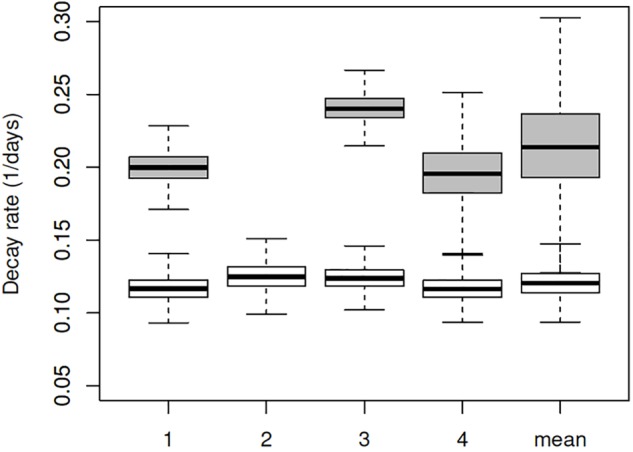
Box plot of decay rates estimated for all four experiments separately (1–4) and combined. White boxes represent the decay rates for RNA copies, and the gray boxes for TCID_50_. Plots show median (bar), quartiles (box), and 95% predictive range (error bars).

Estimated decay rates were calculated for each experiment separately (**Figure 3**), and despite some differences observed in the water temperature and salinity, values obtained are comparable (**Table 3**). The mean decay of 0.120 log_10_ units per day (95% CI 0.10–0.15 per day) corresponds to a decay rate for detectable viruses of 3.6 log_10_ units in 1 month (30 days) and a half-life of 2.5 days (95% CI 2.0–3.1 days).

**Table 3:** Initial numbers and decay rates of Tulane virus (TV) as measured by *r*RT-PCR (genome copies) and infectivity assay (TCID_50_), as estimated by the two-level Bayesian regression model.

Experiment	Concentration^1^			Decay rate^2^		
		
	Median	*P*_2.5_	*P*_97.5_	Median	*P*_2.5_	*P*_97.5_
**RT-PCR (Genome Copies)**					
A	1.54 × 10^6^	0.84 × 10^6^	2.74 × 10^6^	0.117	0.100	0.136
B	1.55 × 10^6^	0.80 × 10^6^	2.83 × 10^6^	0.125	0.105	0.145
C	2.38 × 10^6^	1.35 × 10^6^	4.28 × 10^6^	0.124	0.108	0.140
D	2.11 × 10^6^	1.15 × 10^6^	4.00 × 10^6^	0.116	0.100	0.133
Overall	1.86 × 10^6^	0.85 × 10^6^	3.71 × 10^6^	0.121	0.098	0.146
**Infectious virus (TCID50)**					
A	6.57 × 10^3^	4.77 × 10^3^	9.03 × 10^3^	0.200	0.179	0.221
B^3^	NA	NA	NA	NA	NA	NA
C	1.32 × 10^4^	9.50 × 10^3^	1.86 × 10^4^	0.240	0.221	0.260
D	1.34 × 10^4^	8.34 × 10^3^	2.18 × 10^4^	0.196	0.153	0.237
Overall	9.86 × 10^3^	2.94 × 10^3^	3.40 × 10^4^	0.214	0.147	0.325


**The 97.5th and 2.5th percentile values are also shown. ^*1*^Concentrations are expressed as RNAc/g of DT or TCID_*50*_/g of DT with median estimates and 95% range calculated based on the four extraction results obtained. ^*2*^Decay rates, expressed as decrease in log_*10*_ numbers of RNAc or TCID_*50*_ per day, in each of the four experiments, and estimated mean decay rate over all experiments combined (median estimates and 95% range). ^*3*^TCID_*50*_ data from experiment B discarded as freezing these samples prior to analysis clearly resulted in a reduction in infectious virus. NA, nucleic acid*.*

### Detection of Infectious TV

Genomic concentrations of inoculum prepared for infectivity showed a decrease of about 50%. Within the error range of the quantification by *r*RT-PCR, and thus all these inoculum were used for infectivity tests. Infectious TV was detected for up to 21 days in three experiments, and for 14 days in experiment D (**Table 2**). Experiments A and C showed similar decreases in infectious virus, with quantification reaching the limit of detection after 2 weeks, and after 1 week for experiment D. For this last experiment, infectious TV was detected only for 14 days presumably because of a slight increase in water temperature. The fastest decrease in infectious TV for experiment B was probably caused by freezing the samples at -20°C before analysis, and therefore, the results were not included in the data analysis.

When added to the Bayesian regression model, the infectivity data allowed the fraction of infectious virus to be estimated (compared to the total number of genome copies). Assuming exponential decay of the infectious fraction, the estimated initial numbers and decay rates are shown in the lower part of **Table 3** and **Figure 3**. The decay rate of infectious virus is estimated at 0.214 log_10_ units per day (95% CI 0.147–0.325 per day), which corresponds to a 6.42 log_10_ unit decrease in 1 month (30 days). The corresponding half-life is 1.4 days (95% CI 0.9–2.0 days).

## Discussion

To infect their human host, HuNoVs required to bind to carbohydrates of the histo-blood group antigen (HBGA) family (Atmar et al., 2014; Le Pendu et al., 2014). Binding of HuNoV to similar ligands was demonstrated in oyster digestive tissues (Le Guyader et al., 2006), impacting bioaccumulation efficiency, with seasonal differences shown for some strains (Maalouf et al., 2010, 2011). In addition to HuNoVs, within the *Caliciviridae* family, members of the lagovirus and recovirus genera also recognize HBGAs of their respective hosts (Farkas et al., 2008, 2014; Le Pendu et al., 2014). Yet, among these viruses only some ReCVs such as TV are cultivable to high titers in susceptible cells and present the same stability as HuNoVs in seawater, as demonstrated by genome detection (Drouaz et al., 2015). Moreover, TV and Norwalk virus have similar tissue distributions and half-lives in oysters, confirming that TV is an appropriate surrogate for studying HuNoV behavior in shellfish (Drouaz et al., 2015). Infectious TV particles detected after the heat treatment of eastern oysters (*Crassostrea virginica*) confirmed its higher stability compared to murine norovirus (MNV-1) (Araud et al., 2016). Additionally, TV treated at 70°C lost 100% of infectivity (84% at 56°C for 2 min) (Wang et al., 2014), a rate comparable to the inactivation of GII.3 and GII.4 as demonstrated on human enteroids (Ettayebi et al., 2016), and this confirmed its interest as a HuNoV surrogate (Cromeans et al., 2014).

Infectivity studies that can be undertaken with cell culture-adapted ReCVs are therefore of utmost importance for shellfish. Detecting infectious viral particles in oysters requires methods of virus recovery that do not damage the virus capsid, and eliminate the cytotoxic impact of the oyster matrix on cell cultures. Long before the development of molecular techniques, sensitive methods were developed to detect infectious enterovirus from various environmental samples, including shellfish (Metcalf et al., 1980). PEG was proved an effective concentration method to recover a variety of infectious human enteric viruses such as hepatitis A virus, rotavirus and poliovirus from oyster tissues (Lewis and Metcalf, 1988). Later on, PEG was also used for molecular detection (Atmar et al., 1995), and to detect HuNoV in shellfish-related outbreaks (Le Guyader et al., 2008, 2010). We reasoned that the use of identical initial recovery steps for both genome and infectious virus detection would enable the results to be more meaningfully compared, and thus, we decided to focus on the PEG method. After several failed attempts, some purification steps were added to reduce the toxicity to the cells, allowing the successful detection of infectious virus while minimizing the lost of particles. This PEG method was then applied to evaluate the decrease of genome copies and infectious particles in contaminated oysters kept in a scientific farm to mimic producer’s farm environment. Another approach using direct elution with no centration step was reported efficient but need higher concentrations (Araud et al., 2016).

Quantification of genome copies in oyster DTs was achieved using *r*RT-PCR. Despite the application of controls as recommended in the ISO/TS 16216-1 method (International Organization for Standardization, 2013), questions have been raised regarding the accuracy and variability in RNA quantification using *r*RT-PCR between laboratories, and error propagation through the use of reference materials. Analysis of quantitative PCR data is not a trivial problem: aside from uncertainties in measured *C*_T_-values, as revealed by differences between replicate measurements, the translation of *C*_T_-values into genome copies causes additional uncertainty. Indeed, the standard curve, as defined by the same (uncertain) *C*_T_ data, must be inverted to translate *C*_T_-values into genome copy number estimates. Quality criteria to validate these curves, and triplicate sample extractions provide additional assurances regarding quantification (Drouaz et al., 2015; Le Mennec et al., 2017). In order to use all information provided by this hierarchical setup, and estimate the resulting uncertainty, appropriate statistical models considering all sources of uncertainty are needed. The Bayesian approach, nesting the sample data into the standard curve regression model, with *C*_T_-values known and genome copy numbers missing, considers the error associated to the *C*_T_-value of the sample. Running the Bayesian model then produces estimates for missing data, or rather a sample from their joint posterior distribution. Even high *C*_T_-values above the cutoff for quantification, e.g., *C*_T_-values greater than 40, can be included as censored observations, with the mere effect of inflated error estimates (**Figure 2**).

Using this approach, we observed a mean decay rate value of 0.121 log_10_ RNAc per day, corresponding to a half-life of 2.0–3.1 days, shorter but in the same range, than our previous estimate of 4.56 days (Drouaz et al., 2015). The accuracy of the quantification method applied here and other factors such as the use of different oyster batches and variable water content (nutrients, salinity, water temperature…) may explain this difference. Regarding the temperature parameter, the same decay rate was observed for the experiment performed with water temperature around 17°C compared to the others, consistent with earlier studies showing no significant decay of HuNoV at this temperature (Schwab et al., 1998; McLeod et al., 2009). However, it would interesting to further evaluate the impact of this parameter by repeating experiments in various condition. The model used, estimating variability associated to the measurement for low concentrations, allows us to conclude that the concentration variability observed in oyster tissues after 1 month is likely linked to inter-animal variability rather than to quantification errors, as PCR variation at this concentration was lower than the overall variation observed. Oysters through their physiological activity may eliminate the viral particles at variable individual rates. Future developments may include a more comprehensive regression model including covariates like oyster batch, water temperature or salinity.

The major result of this study is the demonstration that a ReCV, a virus closely related to the *Norovirus* genus, remains infectious for 3 weeks in oysters immersed in natural running seawater. The mean decay rate for infectious particles was 0.214 log_10_ TCID_50_ per day compared to 0.121 log_10_ RNAc per day, thus, the long-lasting question of whether infectious particles decrease at a faster rate than genome copies is partly answered. Reports on the detection of infectious viruses from shellfish are rare, making these data original. Although the infectious virus titer decreases faster than the concentration in viral genome copies, it is only of a factor two. Indeed, infectious particles were still present after 3 weeks confirming observations made following illness outbreaks in France, from which we concluded that the contamination events occurred 2 or 3 weeks before marketing and consumption of contaminated oysters (Polo et al., 2016; Le Mennec et al., 2017). Hepatitis A cases in the Netherlands were also linked to contamination events occurring several weeks before consumption (Boxman et al., 2016). Our study also underscores the need for further method refinements to improve the sensitivity and to increase the sample size. Such an approach will be beneficial both for genome detection as it will move the *C*_T_-values into an area where the PCR gives more reliable results, and for the detection of infectious viruses, as after 3 weeks some infectious particles were detected, albeit below the limit of quantification. Being able to detect infectious Tulane virus is a step forward toward a better understanding of viral persistence in oysters. This model could be used to validate potential treatments such as longer depuration time, but also other methods such as *in situ* capture using pig mucin or HBGA (Tian et al., 2017; Randazzo et al., 2018), keeping in mind the ultimate goal to compare with infectious HuNoV using the model based on enteroids (Ettayebi et al., 2016).

Our results, based on genome detection and infectious particle detection of the Tulane virus, member of the *Caliciviridae* family, show the long persistence of infectious virus, in shellfish and highlight the need to date more precisely contamination events to estimate the infectious risk associated with detection of the viral genome. This strengthens the recommendation for closure of production areas for several weeks after a contamination event, such as the 4-week closure applied in France. Such data will be useful for local authorities to manage producing areas implicated in outbreaks, and thus to protect shellfish consumers.

## Author Contributions

DP and JS performed the experiments and analysis. PT designed the model and analyzed the data. VB was in charge of the shellfish care and related analysis. FLG designed the study and wrote the manuscript.

## Conflict of Interest Statement

The authors declare that the research was conducted in the absence of any commercial or financial relationships that could be construed as a potential conflict of interest.
