# Supplementary material for: Infectivity and RNA Persistence of a Norovirus Surrogate, the Tulane Virus, in Oysters
Source: Front Microbiol. 2018 Apr 12;9:716. doi: 10.3389/fmicb.2018.00716 (PMC5906594; doi:10.3389/fmicb.2018.00716)
Supplement: Supplementary file 1 [file Data_Sheet_1.pdf]

Technical details of statistical methods

Standard curve: Linear regression

When the concentration of virus (genomes) in the suspension is c, a volume V of that suspension represents an expected number of $x = cV$ genome copies, leading to a C_T value y. When x is the log of the number of genome copies then the expected value $E(x) = log(cV) = log(c) + log(V)$. The expected value $E(y) = \mu_y = a + bE(x) = a + blog(cV)$. Assume the measurement error in y is normally distributed with variance σ_y^2, then the likelihood of observing C_T values $\boldsymbol{Y} = (Y_1, Y_2, \ldots, Y_N)$ at log numbers of genome copies $\boldsymbol{X} = (X_1, X_2, \ldots, X_N)$ is

$$\ell(a, b, \sigma_y) = \prod_{n=1}^{N} N(Y_n | a + bX_n, \sigma_y)$$

where $N()$ is the density of the normal distribution. This likelihood allows estimation of a and b, and the error term σ_y. Given N (number of genome copies), the error in C_T is calculated. However, for a given (set of) observation(s) of the C_T value y we need to know the error in the estimated numbers of viruses. This is the inverse problem: the C_T value is known, and the numbers of genome copies must be estimated (Halperin, 1970; Hoadley, 1970).

Inverse regression

This problem is easy in a Bayesian analysis. Assuming prior densities for (a, b) and σ_y

$$f(a, b) = MVN(a, b | \mu_{a,b}, \Sigma_{a,b} \text{ and } g(1/\sigma_y^2 \sim \Gamma(r_\sigma, \lambda_\sigma$$

a posterior density is obtained

$$h(a, b, \sigma_y = \ell(a, b, \sigma_y) f(a, b) g(1/\sigma_y^2)$$

This posterior can be used to estimate a, b, and σ_y , which produces the same result as the standard linear regression above (provided uninformed priors). The advantage of a Bayesian approach becomes clear when there is a sample with known C_T: Y_s and unknown log number of genome copies x_s. Its likelihood

$$\ell_\text{s}(x_\text{s}, a, b, \sigma_y) = N(Y_\text{s} | a + bx_\text{s}, \sigma_y)$$

and prior for the unknown x_s is $\phi(x_\text{s}) = N(x_\text{s} | \mu_\text{s}, \sigma_\text{s})$. The joint posterior probability, together with the contribution from the standard curve, is

$$h(x_\text{s}, a, b, \sigma_y) = \ell(a, b, \sigma_y) f(a, b) g(1/\sigma_y^2) \ell_\text{s}(x_\text{s}, a, b, \sigma_y) \phi(x_\text{s})$$

which can be used to jointly estimate (a, b), and y, and the unknown number of genome copies x_s.

Virus decay curves

The decrease in virus numbers is modeled as an exponential decay process, with the logarithm of the (expected) number of genome copies $x = \log(cV)$ decaying with time t as

$$x(t) = x_0 - \frac{t}{\lambda_{\text{gc}}}$$

where λ_{gc} is the time constant for the decay. If the logarithm of the fraction infectious virus z decreases over time as

$$z(t) = z_0 - \frac{t}{\lambda_{\text{inf}}}$$

the logarithm of numbers of infectious viruses decay as

$$x(t) - z(t) = (x_0 - z_0) - t\left(\frac{1}{\lambda_{\text{gc}}} + \frac{1}{\lambda_{\text{inf}}}\right)$$

Parameter estimation

All parameters were estimated in a Bayesian hierarchical framework, estimating the pairs $(x{-}0, \lambda_{\text{gc}})$ and $(z_0, \lambda_{\text{inf}})$ by experiment, and defining flat (hyper–)distributions for the decay parameters λ_{gc} and λ_{inf}, assuming both $\log(\lambda_{\text{gc}})$ and $\log(\lambda_{\text{inf}})$ normally distributed.
The model was implemented in JAGS (v4.2.0), with burnin 10,000 samples and 3 chains run in parallel of 10,000 samples each, thinning the resulting Markov chains down to 3 times 1,000 samples, checking convergence. Model code is available upon request (PT).

References

Halperin M. On inverse estimation in linear regression. Technometrics 1970; 12(4):727–736.

Hoadley B. A Bayesian look at inverse linear regression. Journal of the American Statistical Association 1970;65(329):356–369.
